# Supplementary material for: Activities Carried Out during the First COVID-19 Lockdown by Italian Citizens
Source: Int J Environ Res Public Health. 2023 Feb 22;20(5):3906. doi: 10.3390/ijerph20053906 (PMC10001561; doi:10.3390/ijerph20053906)
Supplement: Supplementary file 1 [file ijerph-20-03906-s001.zip › ijerph-2190372-supplementary.pdf]

SM1. QUESTIONNAIRE ON LIFESTYLE CHANGES IN THE GENERAL POPULATION OF THE PROVINCE OF REGGIO EMILIA FOLLOWING THE COVID-19 LOCKDOWN

SOCIODEMOGRAPHIC FACTORS

1. **Sex**

F	M

2. **Age**

18-23	24-29	30-35	36-41	42-47	48-53	54-59	60-65	66-71	>71

3. **CITY OF RESIDENCE: ___________________**

4. **Postal code: _________________**

5. **Citizenship:**
- Italian
- Foreign

6. **Marital status:**
- Married
- Cohabitation
- Unmarried
- Widowed
- Divorced
- Separated

7. **Education level**
- None
- Primary school
- Middle school
- High school
- University degree
- Post-university degree

8. **Current home size**
- < 50 m^2
- 50 - 100 m^2
- > 100 m^2

9. **Does your home have any outdoor areas?**
- Garden
- Terrace
- Balcony
- Other
- None

10. **Do you have any children?**

- No
- Yes, at least one child under the age of 12
- Yes, all children over the age of 12

11. **Who do you live with? (multiple choice)**

- Alone
- With partner
- With child/children
- With parents
- With a family member with a serious disability (child/parent)
- With several family members with serious disabilities (children or parents)
- With other relatives or cohabitants

12. **In total, how many people live with you (excluding yourself)? :________**

13. **Are you in isolation after testing positive for COVID-19?**

- Yes
- No

14. **If so, what kind of isolation?**

- Own home
- Hotel
- Rems (COVID-19 residential facility)
- Other temporary home

WORK-RELATED FACTORS

15. **Before the COVID-19 emergency, what was your employment status?**

- Employee in the public sector
- Employee in the private sector
- Self-employed
- Student
- Retired
- Unemployed
- Housewife
- Other (specify): ____________________

16. **Before the COVID-19 emergency, where did you work?**

- At my workplace
- From home
- Both from home and at my workplace

17. **Current employment status**

- Continue to work
- On vacation leave
- Leave of absence
- Unemployment insurance
- Business closed due to the COVID-19 emergency
- Other

18. **If you work or study, how do you do these activities?**

- My job has been suspended
- I go to work
- From home

- I work both from home and at my workplace
- I follow class lectures from home

19. **Are you currently experiencing economic problems?**
    - No difficulties
    - Some difficulties
    - Many difficulties

USE OF DIGITAL DEVICES DATA

20. **Do you currently have a suitable Internet connection?**

- Yes
- No
- I don't know

21. **Do you currently have any IT devices, such as a computer, tablet, and/ or smartphone, and a printer?**

- Yes
- No
- I don't know

22. **How do you evaluate your computer literacy?**
- Poor
- Sufficient
- Good
- Excellent

LIFESTYLE CHANGES, ANTRHOPOMETRIC DATA AND HEALTH STATUS

23. **Weight:____**
24. **Height:______**
25. **Do you have any disease-related fee exemptions?**
- Yes
- No

26. **If exempt, for which disease? (optional question)**

27. **Before the COVID-19 emergency, how did you consider yourself?**
- Active (a person who has a physically demanding job that requires considerable physical effort and/or 30 minutes of moderate physical activity at least 5 days a week and/or intense activity for more than 20 minutes for at least 3 days a week)
- Partially active (a person who does not do a physically demanding job but who does some physical activity in his/ her free time)
- Sedentary (a person who neither has a physically demanding job nor does any physical activity in his/ her free time)

28. **If you were active or partially active, how many days per week?**
- 1-2 days / week
- 3-4 days / week
- > 4 days / week

29. **Currently, how do you consider yourself?**

- Active (a person who has a physically demanding job that requires considerable physical effort and/or 30 minutes of moderate physical activity at least 5 days a week and/or intense activity for more than 20 minutes for at least 3 days a week)
- Partially active (a person who does not do a physically demanding job but who does some physical activity in his/ her free time)
- Sedentary (a person who neither has a physically demanding job nor does any physical activity in his/ her free time)

30. **If you are active or partially active, how many days per week?**

- 1-2 days / week
- 3-4 days / week
- > 4 days / week

31. **What kind of physical activity do you currently do?**

- Treadmill / running / walking / stairs
- Stationary bike / bicycling
- Pilates / Yoga / stretching / free body / gym
- Gardening
- Other:____________________

32. **What kind of IT do you use to perform physical activity at home?**

- Group or one-to-one video chat
- Online courses/ guides
- Applications on smartphones, tablets, computer
- Programs from your trainer
- Alone
- Other

33. **Has your diet changed (improve/ worsen in terms of the consumption of some foods, meal times, weight control)?**

- Yes
- No
- I don't know

34. **If so, what changes have occurred? (multiple choice)**

- I have add snacks, sweets, carbonated drinks
- I eat more abundant main meals (breakfast and/or lunch and/or dinner)
- I eat at more regular times
- I pay more attention to eating healthier (i.e. quality and/or variety and/or meal cooking)
- I eat more prepackaged foods
- Before COVID-19, I was forced to often eat out

35. **Before the COVID-19 emergency, how many portions of fruit or vegetables did you eat daily?**

(portion means a quantity of raw fruit or vegetables that can be held in the palm of your hand, or half a plate of cooked vegetables)

- No portion of fruit or vegetables
- 1-2 portions / day
- 3-4 portions / day
- 5-6 portions / day
- > 6 portions / day

36. **Following the COVID-19 emergency, how has your consumption of fruit and vegetables changed?**

- Increased

- Decreased
- Unchanged
- I don’t eat fruit or vegetables

37. **Do you currently consider your diet healthy?**

- Yes
- No
- I don’t know

38. Before the COVID-19 emergency, how many alcoholic drinks did you consume? (The alcoholic unit (UA) corresponds to 12 grams of ethanol, an amount approximately contained in a can of beer (330 ml at 4.5 °), a glass of wine (125 ml at 12 °), a glass of liqueur (40 ml at 40 °), or an aperitif (80 ml at 38 °).

- None
- < 2 alcoholic unit (UA) on average per day
- ≥2 alcoholic unit (UA) on average per day

39. **Following the COVID-19 emergency, how has your consumption of alcohol changed?**

- Increased
- Decreased
- Unchanged
- I don't drink alcohol

40. **Before the emergency COVID-19, how did you consider yourself in terms of smoking?**

- Non-smoker (a person who claims to have smoked less than 100 cigarettes in his/ her life (5 packs of 20) and is not currently a smoker)
- Smoker (a person who claims to have smoked at least 100 cigarettes in his/ her life (5 packs of 20) and to be a smoker at the time of the interview or to have stopped smoking less than 6 months ago)
- Occasional smoker (a person who declares to not smoke every day)
- Daily smoker (a person who claims to smoke at least one cigarette every day).
- Ex-smoker (a person who claims to have smoked at least 100 cigarettes in his/ her life (5 packs of 20), to NOT be a smoker at the time of the interview, and to have stopped smoking at least 6 months ago)
- Abstention smoker (a person who claims to have quit smoking less than 6 months ago)

41. **Following the COVID-19 emergency, how has your consumption of cigarettes changed?**

- Increased
- Decreased
- Unchanged
- I’m not a smoker

42. **Have your sleep habits changed during lockdown (sleep quantity and regularity)?**

- Yes
- No
- I don’t know

43. **How do you consider your sleep quality now?**

- Very good
- Good
- Not very good
- Not good at all

44. **What new activities you are doing now that you did not do before the COVID-19 lockdown?**

- Reading
- Resting
- Cooking
- Watching TV
- Using social media
- Dedicating time to family activities
- Gardening / housecleaning
- Volunteer work
- None
- Other: ___________________

45. **In the future, are you going to maintain your acquired lifestyle habits or return to your previous behaviors?**

**a) Physical activity**	maintain	return	I don't know/ unchanged
**b) Eating habits**	maintain	return	I don't know/ unchanged
**c) Smoking, drinking habits**	maintain	return	I don't know/ unchanged
**d) Sleep habits**	maintain	return	I don't know/ unchanged
**e) Leisure activities**	maintain	return	I don't know/ unchanged

USE OF SUPPORT SERVICES OF THE TERRITORIAL NETWORK DATA

46. Have you used/are you using any of the support made available by the municipality, the nonprofit volunteer associations, and/ or the Local Health Authority? (multiple choice)

- Grocery delivery
- Home delivery of drugs
- Information hotline
- Mental health hotline
- Economic aid, e.g., food vouchers
- Area animal care facilities
- Activation of social services
- Other: ___________________

47. **What kind of support would you have liked in order to deal with this situation, but which was not available?** (Open question)

____

____

48. **What/ who helped you to overcome the challenges of lockdown? (multiple choice)**

- family or friends
- volunteers / neighbors
- healthcare professionals
- the municipality or local associations
- leisure activities (i.e., reading, gardening, etc.)
- continuing to work
- optimistic attitude
- physical activity
- drinking and/ or smoking

- taking care of pets
- salary
- drugs
- other

EMOTIONAL STATE

49. At the moment, are you feeling:

**Nervous**	1. Not at all	2. A little	3. Quite	4. A lot
**Upset**	1. Not at all	2. A little	3. Quite	4. A lot
**Worried**	1. Not at all	2. A little	3. Quite	4. A lot
**Afraid**	1. Not at all	2. A little	3. Quite	4. A lot
**Lonely**	1. Not at all	2. A little	3. Quite	4. A lot
**Uncertain about the future**	1. Not at all	2. A little	3. Quite	4. A lot

SM2. STROBE Statement—Checklist of items that should be included in reports of ***cross-sectional studies***

	Item No	Recommendation	Page No
**Title and abstract**	1	(*a*) Indicate the study's design with a commonly used term in the title or the abstract	1
		(*b*) Provide in the abstract an informative and balanced summary of what was done and what was found	1
**Introduction**			
Background/rationale	2	Explain the scientific background and rationale for the investigation being reported	1-3
Objectives	3	State specific objectives, including any prespecified hypotheses	3
**Methods**			
Study design	4	Present key elements of study design early in the paper	3
Setting	5	Describe the setting, locations, and relevant dates, including periods of recruitment, exposure, follow-up, and data collection	3
Participants	6	(*a*) Give the eligibility criteria, and the sources and methods of selection of participants	3
Variables	7	Clearly define all outcomes, exposures, predictors, potential confounders, and effect modifiers. Give diagnostic criteria, if applicable	3,4
Data sources/ measurement	8*	For each variable of interest, give sources of data and details of methods of assessment (measurement). Describe comparability of assessment methods if there is more than one group	3,4
Bias	9	Describe any efforts to address potential sources of bias	3,4
Study size	10	Explain how the study size was arrived at	NA
Quantitative variables	11	Explain how quantitative variables were handled in the analyses. If applicable, describe which groupings were chosen and why	3,4
Statistical methods	12	(*a*) Describe all statistical methods, including those used to control for confounding	4
		(*b*) Describe any methods used to examine subgroups and interactions	NA
		(*c*) Explain how missing data were addressed	NA
		(*d*) If applicable, describe analytical methods taking account of sampling strategy	NA
		(*e*) Describe any sensitivity analyses	NA
**Results**			
Participants	13*	(a) Report numbers of individuals at each stage of study—eg numbers potentially eligible, examined for eligibility, confirmed eligible, included in the study, completing follow-up, and analysed	4-13

		(b) Give reasons for non-participation at each stage	NA
		(c) Consider use of a flow diagram	NA
Descriptive data	14*	(a) Give characteristics of study participants (eg demographic, clinical, social) and information on exposures and potential confounders	4-13, Table 1
		(b) Indicate number of participants with missing data for each variable of interest	Table 1
Outcome data	15*	Report numbers of outcome events or summary measures	4-13, Table 1
Main results	16	(*a*) Give unadjusted estimates and, if applicable, confounder-adjusted estimates and their precision (eg, 95% confidence interval). Make clear which confounders were adjusted for and why they were included	4-13, Table 1
		(*b*) Report category boundaries when continuous variables were categorized	NA
		(*c*) If relevant, consider translating estimates of relative risk into absolute risk for a meaningful time period	NA
Other analyses	17	Report other analyses done—eg analyses of subgroups and interactions, and sensitivity analyses	NA
**Discussion**			
Key results	18	Summarise key results with reference to study objectives	13-16
Limitations	19	Discuss limitations of the study, taking into account sources of potential bias or imprecision. Discuss both direction and magnitude of any potential bias	15,16
Interpretation	20	Give a cautious overall interpretation of results considering objectives, limitations, multiplicity of analyses, results from similar studies, and other relevant evidence	13-16
Generalisability	21	Discuss the generalisability (external validity) of the study results	16
**Other information**			
Funding	22	Give the source of funding and the role of the funders for the present study and, if applicable, for the original study on which the present article is based	16

*Give information separately for exposed and unexposed groups.

Note: An Explanation and Elaboration article discusses each checklist item and gives methodological background and published examples of transparent reporting. The STROBE checklist is best used in conjunction with this article (freely available on the Web sites of PLoS Medicine at http://www.plosmedicine.org/, Annals of Internal Medicine at http://www.annals.org/, and Epidemiology at http://www.epidem.com/). Information on the STROBE Initiative is available at www.strobe-statement.org.
